# P01-044 – Uncommon manifestations of familial Mediterranean

**DOI:** 10.1186/1546-0096-11-S1-A47

**Published:** 2013-11-08

**Authors:** A Beglaryan, K Ginosyan, A Ayvazyan

**Affiliations:** 1 Rheumatology, Center of family planning and sexual health, Yerevan, Armenia; 2 Internal Diseases, Yerevan State Medical University, Yerevan, Armenia

## Introduction

Familial Mediterranean Fever (FMF) is the most common autoinflammatory disease characterized by recurrent self-limited attacks of fever accompanying with peritonitis, pleuritis or arthritis. The testing of MEFV gene expanded the frame of various clinical manifestations of FMF.

## Objectives

The aim of this study is to determine systemic uncommon non-classical manifestations of FMF and reveal the possible association with autoimmune diseases.

## Methods

We examined 50 patients (27 male, 23 female) with FMF. The mean age of patients was 34.7±12.2. FMF was determined clinically and approved by testing of MEFV gene. To reveal systemic manifestations of FMF investigation of all organ-systems was carried out.

## Results

Classical symptoms of FMF: abdominalgy, thoracalgy, arthralgy with recurrent fever and pleuritis, splenomegaly, hepatomegaly were seen in almost all patients. Monoarthritis was met in 36 (72%) and polyarthritis in 8(16%) patients. 40(80%) patients developed spondyloarthrtitis, in 11 cases (22%) unilateral sacroiliitis and in 29(58%) bilateral sacroiliitis was observed. All patients with sacroiliitis fulfilled the classification criteria of the European Spondyloarthropathy Study Group for the diagnosis of seronegative spondyloarthropathy. 10(25%) patients of 40 that having sacroiliitis developed significant limitation of lumbar motion, which was assessed by Schober’s test (1-2 cm), had bilateral sacroiliits grade III-IV and fulfilled the modified New York criteria for ankylosing spondylitis. HLA B-27 was examined in 15 patients with symptoms of spondyloarthropathy. In 7 patients it was negative and in 8-positive. In19 (38%) from 50 patients coxarthrosis was revealed and 2 patients underwent total endoprothesis of hips. Skin involvement also was observed during the observation: 4(8%) of them developed erythema similar “butterfly” rash, livedo reticularis and photosensitivity with high titer of circulating immune complexes, ANA and anti-dsDNA antibodies like systemic lupus erythematosus, 1 patient had hemorrhagic rash on legs with developing of hemorrhagic vasculitis. In 1 patient trophic ulcers, miscarriage were developed with high titer of anticardiolopin autoantibodies as in classic antiphospholipid syndrome. Scleroderma-like syndrome was developed in 1 patient with Raynaud’s phenomenon and skin induration of wrists and face and pneumofibrosis. Also panniculitis (1 patient), aphthae (2 patients), angiorethinopathy(2 patients), mononeyropathy and polyneyropathy ( 2 patients respectively), pneumonitis (4 patients), xerophthalmia (1 patient) like Sjogren’s syndrome were observed. The prevalent mutation of MEFV gene was M694V- in 38patients (79.1%), from which 8(16.7%) were homozygote and 14(29%) were heterozygote (M694V/N).

## Conclusion

FMF may have systemic manifestations of autoimmune diseases. It may be due to vascular involvement especially in accompaning amyloidosis cases. The peculiarity of ankylosing spondylitis-like syndrome in FMF is its independent existence from carrying HLA B-27 antigen.

## Disclosure of interest

None declared.

